# Laser Assisted (Er:YAG and Nd:YAG) Minimally Invasive Peri-Odontal Surgery in the Treatment of Intrabony Defects—A 12-Month Observational Randomized Clinical Trial

**DOI:** 10.3390/bioengineering12091002

**Published:** 2025-09-22

**Authors:** Anna Skurska, Ewa Dolińska, Robert Milewski, Małgorzata Pietruska

**Affiliations:** 1 Department of Integrated Dentistry, Medical University of Bialystok, ul. M. Skłodowskiej-Curie 24A, 15-276 Bialystok, Poland; 2 Department of Periodontal and Oral Mucosa Diseases, Medical University of Bialystok, ul. Waszyngtona 13, 15-269 Bialystok, Poland; ewa.dolinska@umb.edu.pl (E.D.); mpietruska@wp.pl (M.P.); 3 Department of Biostatistics and Medical Informatics, Medical University of Bialystok, ul. Szpitalna 37, 15-295 Bialystok, Poland; robert.milewski@umb.edu.pl

**Keywords:** periodontal regeneration, MIST, periodontal intrabony defects, Er:YAG laser, Nd:YAG laser

## Abstract

**Objectives**: The objective of our study was to assess changes in the clinical and radiological parameters after modified minimally invasive surgical technique (M-MIST) in the treatment of intrabony periodontal defects with additional Er:YAG and Nd:YAG laser applications. **Methods**: Thirty-eight patients, each presenting with a single vertical defect, were randomly assigned to either the test (M-MIST+Er:YAG+Nd:YAG) or the control group (M-MIST). Probing depth (PD) reduction, clinical attachment level (CAL) gain (primary outcomes of the study) were assessed prior to therapy and after 12 months following the surgical procedure. **Results**: Both methods led to statistically significant improvements in clinical (PD reduction and CAL gain) and radiological parameters. No statistical differences were observed between the groups at any time point assessed. At 12 months postoperatively, radiographic defect depth reduction was very similar in both groups. The radiographic defect width decrease was more pronounced in the control group. **Conclusions**: Results indicate that use of Er:YAG and Nd:YAG lasers combined with the M-MIST procedure and the conventional M-MIST procedure provides comparable clinical and radiological treatment outcomes.

## 1. Introduction

Periodontitis is a progressive, destructive disease of the tooth suspension apparatus. It affects the gums, periodontium, root cement, and alveolar bone [1]. The clinical picture of the disease shows loss of connective tissue attachment, periodontal pocket formation, and tooth loosening, while X-rays demonstrate horizontal and vertical alveolar bone loss.

Bacterial biofilm is considered the main etiological factor causing gingivitis and leading to the destruction of periodontal tissues [2].

The nature of the periodontal changes determines the therapeutic and preventive measures [3,4]. They focus on anti-infective action and reconstruction of the teeth’s surrounding structures [5,6,7].

The aim of periodontal regeneration is to restore tissues lost due to periodontal disease. Various treatment methods have been described in the literature, including biomodification of the root surface with enamel matrix derivative (EMD), guided tissue regeneration (GTR), and the use of growth factors using appropriate incisions and flaps [7,8,9,10]. However, modern reconstructive periodontology focuses on minimally invasive procedures to support natural healing processes [7,8,11].

Conventional surgical approaches, consisting of flaps without papillae preservation, often led to the lack of primary wound closure. This resulted in clot instability and a healing model consisting of epithelial migration into the wound, which can be histologically characterized as repair [11]. The introduction of cuts and flap management, which preserve the interdental tissues, allowed for better protection and stabilization of the treated area [12]. Minimally invasive approaches proved greater amounts of clinical attachment level (CAL) gains and smaller recession [13]. A recognized factor that also influences the effect of reconstructive procedures is precise decontamination of the treatment area [14]. Hence, the search for new solutions/modifications is underway that would help ensure the above-mentioned conditions.

There are not many papers available evaluating the effectiveness of Er:YAG (Er-bium-doped Yttrium Aluminum Garnet) and Nd:YAG (Neodymium-doped Yttrium Aluminum Garnet) utilization in periodontal surgical treatment. Available literature mostly refers to the use of Er:YAG+Nd:YAG in nonsurgical therapy [6]. Findings from a recently published systematic review suggest that the combination of Nd:YAG and Er:YAG lasers may lead to additional clinical improvements in a nonsurgical approach [15]. Lasers are a rather expensive tool, and unfortunately, they are not standard equipment in dental offices. Therefore, they are not the first-choice tool for periodontal treatment. This may explain the limited availability of scientific evidence.

Er:YAG laser emits in the mid-infrared range at a wavelength of 2940 nm. Its target is water or the hydroxide ion and a mineral. It can be used for bacterial reduction, soft-tissue debridement of periodontal diseased tissue, and calculus removal in a nonsurgical approach [16]. Its penetration depth is approximately 5 µm, because it is well absorbed in water [17]. For this reason, the erbium laser is also indicated for calculus removal. Many authors point out the necessity of being careful when dealing with the root surface, because cementum has similar hydration properties to dental calculus [16,17].

The wavelength of Nd:YAG lasers is approximately 1064 nm and is selectively absorbed in areas of inflammation by tissue pigment and blood components [16]. This wavelength is transmitted through water, which explains its deep penetration into healthy soft tissue. When using the Nd:YAG laser, it is important to avoid prolonged contact with the root structure in order to avoid thermal damage [16].

By eliminating calculus and bacteria, Er:YAG and Nd:YAG lasers can create a bio-compatible root surface that may facilitate periodontal healing. Due to the different features, these two lasers have different effects on the hard and soft tissues [18].

It can be assumed that the Er:YAG laser may be an alternative to root biomodification because the irregular surface often produced by the Er:YAG laser may promote only a slight initial inflammatory response while efficiently removing the smear layer, and enhance adhesion and growth of cells as well as blood components [18,19].

Taking into account the above considerations, the objective of our study was to assess changes in the clinical and radiological parameters after minimally invasive surgical treatment of intrabony periodontal defects, M-MIST (Modified Minimally Invasive Surgical Technique) alone or M-MIST with additional Er:YAG and Nd:YAG laser applications, over a twelve-month follow-up period. We assumed changes in clinical parameters (CAL gain and PD reduction) as the primary endpoints and radiological parameters, such as radiological defect depth (RxD) and radiological defect width (RxW), as the secondary endpoints of the study. We formulated the null hypothesis that additional use of laser does not affect clinical parameters, nor does it affect the values of radiological parameters.

## 2. Materials and Methods

### 2.1. Sample Size

The primary study outcome was the change in the CAL. Sample size calculation was performed in order to detect a clinically significant difference of 1.0 mm in the CAL between the two therapeutic procedures. Type I error was set at 0.05 level and power at 0.80, assuming that the standard deviation (SD) was 1.0 mm. The required sample size was calculated to be 16 patients in each group (a total of 32 patients). In anticipation of patient dropout rates of up to 20%, a total of 40 patients were planned for enrollment.

### 2.2. Study Population and Experimental Design

**Figure 1:**
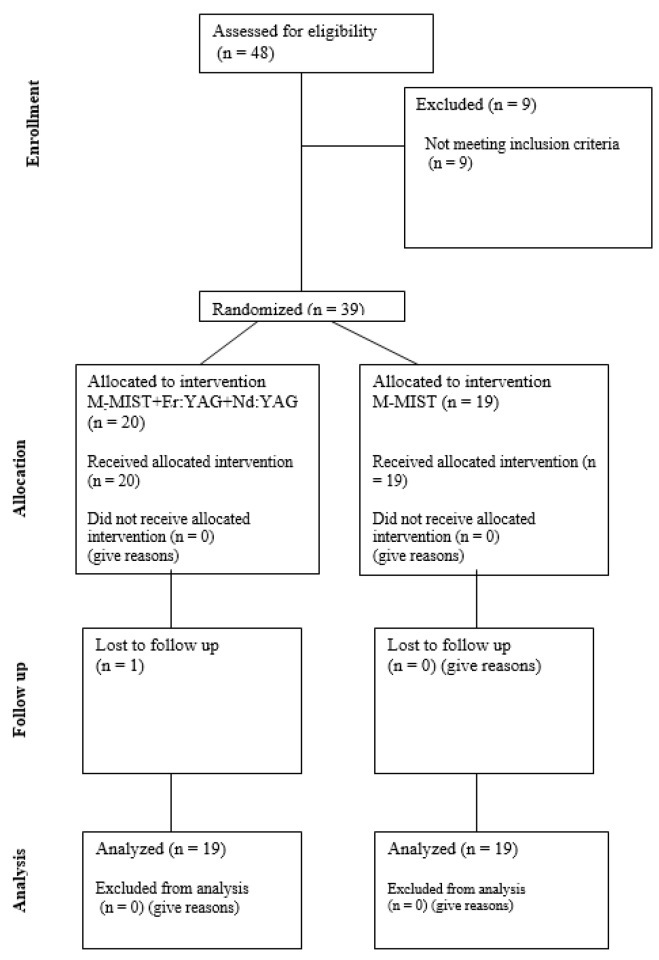
Consort flow diagram.

Thirty-nine non-smoking patients diagnosed with periodontitis stage III (22 women and 17 men) aged 24–73 were included in the study. The study was designed as a single-center, single-blinded, randomized, prospective, controlled clinical trial. Patients were randomly divided into two groups. Allocation of patients to test and control groups was carried out by means of a coin toss. Each participant had only one intrabony defect treated in the project. In the control group (M-MIST), a Modified Minimally Invasive Surgical Technique procedure was performed. In the test group (M-MIST+Er:YAG+Nd:YAG), the Modified Minimally Invasive Surgical Technique was performed with the use of lasers. One patient did not complete the one-year follow-up period and was removed from the analysis. The study consort flow diagram is presented in Figure 1.

The study was performed in accordance with the Helsinki Declaration of 1975, as revised in 2013, and was reviewed and approved by the local ethical committee (Bioethical Committee, Medical University of Bialystok, Nr.: R-I-002-397-2016). Patients were treated in the Department of Periodontal and Oral Mucosa Diseases, Medical University of Bialystok, Poland.

Inclusion criteria for the study were as follows: presence of an intrabony defect, with a pocket depth (PD) ≥ 6 mm and a radiological defect depth of ≥3 mm and width of ≥2 mm; FMPS (Full-mouth Plaque Score) < 20%, FMBOP (Full-mouth Bleeding on Probing) < 20%; over 18 years of age. Patients with general diseases that could affect the healing process, pregnant and breastfeeding women were excluded.

### 2.3. Clinical Examinations, Surgery, and Postoperative Care

The same periodontal probe (UNC 15, Hu-Friedy, Chicago, IL, USA) was used by a masked and calibrated investigator to assess clinical parameters before and 12 months after the surgery. The following clinical parameters were measured for each tooth with an intrabony defect: probing depth (PD), clinical attachment level (CAL), and gingival recession (GR). Each tooth was examined at three points on the buccal and lingual sides (mesial, middle, and distal). The deepest measured point was used for statistical analysis. The cemento-enamel junction (CEJ) or the filling margin was taken as the reference point. FMPS [20] and FMBOP [21] were calculated as a percentage on the four surfaces of each tooth. Also, pre- and postoperative radiographs were taken using the long-cone parallel technique, with the customized bite-positioner. There were two parameters analyzed on the radiographs: defect depth (RxD)—the vertical distance between the bone crest and the site on the root surface at which the periodontium width was normal (in mm), defect width (RxW)—the horizontal distance between the root surface and bone defect margin in the most coronal part of the bone crest (in mm).

**Figure 2:**
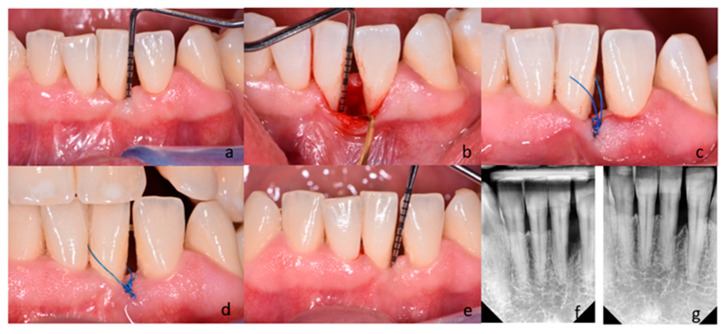
(**a**–**g**) Subject treated with M-MIST (control group); (**a**) Probing pocket depth on the distal surface of tooth 31; (**b**) A surgical site after exposure with MPPT. The depth of intrabony defect; (**c**) Flap reposition and closure with non-absorbable sutures; (**d**) Postoperative view after 2 weeks; (**e**) Probing pocket depth on the surgical site 12 months post-op; (**f**) Radiograph of the bone defect on the distal surface of tooth 31 before treatment; (**g**) Radiograph 12 months after surgery.

Following local anesthesia (Septanest 100, Septodont, Paris, France), intrasulcular incisions and preparation of the mucoperiosteal flap were performed according to the principles of papilla preservation technique [11,22] and minimally invasive techniques [12,23]. In the control group, the debridement of the bone defect was performed with the use of hand instruments (Gracey currettes, Hu-Friedy, Chicago, IL, USA) and ultrasonic devices (EMS Piezon Tip PS, EMS, Nyon, Switzerland) (Figure 2).

**Figure 3:**
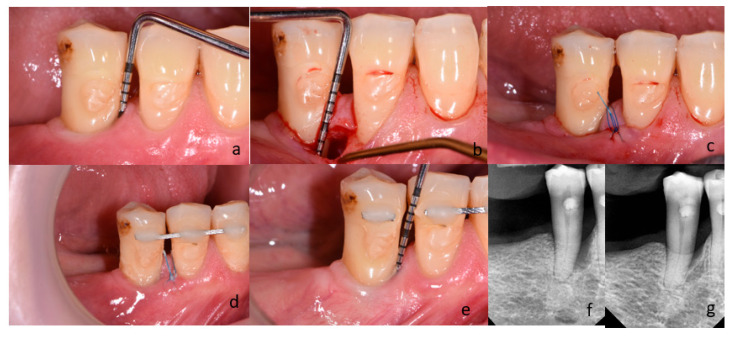
(**a**–**g**) Subject treated with M-MIST+Er:YAG+Nd:YAG (test group); (**a**) Probing pocket depth on mesial surface of tooth 45; (**b**) A surgical site after exposure with MPPT. The depth of intrabony defect; (**c**) Flap reposition and closure with non-absorbable sutures; (**d**) Postoperative view after 2 weeks; (**e**) Probing pocket depth on the surgical site 12 months post-op; (**f**) Radiograph of bone defect on mesial surface of tooth 45 before treatment; (**g**) Radiograph 12 months after surgery.

For the same purposes, a dental laser (Fotona Light Walker AT-S, Ljubljana, Slovenia) was used in the test group. The granulation tissue was removed with the use of Er:YAG laser (3 W, 150 mJ and 20 Hz LP—long pulse 600 µs; handpiece H14-N, sapphire tip), as the root surface debridement (1,6 W, 160 mJ and 10 Hz LP—long pulse 600 µs; handpiece H14-N, sapphire tip). At the end of the surgical procedure, the blood clot formation was performed with the use of Nd:YAG laser (2 W and 20 Hz VLP—very long pulse 1000 µs; handpiece—R21-C3; specialized fiber optic tips) (Figure 3). After completing the debridement of the intrabony defect, the mucoperiosteal flap was repositioned and stabilized by means of vertical modified mattress sutures (Ethilon 5.0, Johnson & Johnson Company, New Brunswick, NJ, USA). If tooth mobility was detected, it was splinted after surgery. Postoperative care consisted of 0.2% chlorhexidine rinses (Eludril, Pierre Fabre Laboratories, Paris, France) twice a day for 2 weeks. Patients were also instructed not to eat hard foods and to avoid vigorous tooth brushing in the surgical area for 2 weeks. Sutures were removed 14 days post-surgery. Recall appointments were scheduled 1, 2, and 4 weeks, and then 3, 6, and 12 months. During recall appointments, supragingival plaque was carefully removed with a brush. Healing and possible complications (flap dehiscence; flap or papillae necrosis, suppuration, inflammation, as well as pain exacerbations) were monitored during the follow-up appointments. Photographs of the surgical area were taken on every visit. The patient and the surgeon were not blinded in the protocol because of technical reasons. Figure 2 and Figure 3 present cases treated in the control and test group, respectively.

### 2.4. Statistical Analysis

In the statistical analysis, the normality of distribution was verified using the Shapiro–Wilk test and the Kolmogorov–Smirnov test with the Lillefors correction. The distribution normality of the analyzed quantitative variables was not found. The Wilcoxon paired test was used to compare dependent variables over time. The nonparametric U Mann–Whitney test was used to compare the quantitative independent variables without normal distribution. Friedman’s test was used for multiple comparisons.

The results were considered statistically significant for *p* < 0.05. Statistica 13.3 (TIBCO Software Inc., Palo Alto, CA, USA) was employed for calculations.

## 3. Results

Screening continued until a total of 40 patients (20 per group) were enrolled. One patient re-signed before treatment without providing any reason, and one patient dropped out because they were moving to another country. All 38 patients completed the 12-month follow-up visits with no further dropouts. No adverse events were reported. Patients were equally distributed between the study and control groups without differences according to age, gender, and intra-surgical defect depth and width. Table 1 depicts the characteristics of patients, teeth, and defects included.

Enrolled patients maintained good oral hygiene, as evidenced by a low full mouth plaque score (FMPS) index throughout the study duration. The baseline mean FMPS in the test group amounted to 10.8% and in the control group 11.6%. These values remained similar during the study period and did not exceed 15%. Full-mouth bleeding on probing (FMBOP) was also low, and it was 10.7% in the test and 10.3% in the control group before treatment. Mean FMBOP was no higher than 15% throughout the study in both groups. After a year, FMBOP was significantly lower in the control group. Indices of oral hygiene and inflammation are depicted in Table 2.

Clinical parameters of surgical sites are presented in Table 3. In both groups, there were significant reductions in PD (probing depth) and gains in CAL (clinical attachment level) at the 12-month follow-up. Gingival recession (GR) did not change in the test group and increased slightly in the control group. There were also no significant differences between groups before treatment and a year post-op. PD reduction was estimated at 2.95 mm in both groups. CAL gain was slightly higher in the test group (3 mm) than in the control group (2.63 mm). Keratinized tissue width was also almost unchanged after surgery, but its stability was more pronounced in the test group, where the difference after a year was only 0.05 mm.

**Figure 4:**
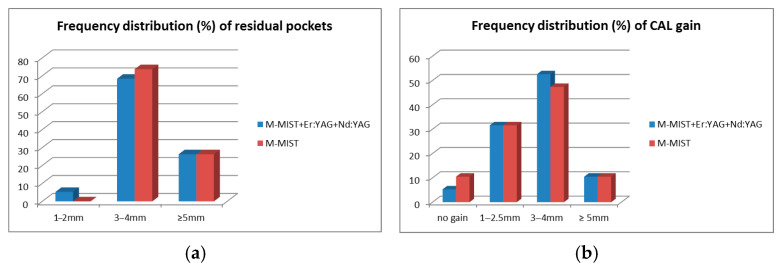
(**a**,**b**) Frequency distribution (%) of residual pockets (**a**) and clinical attachment level gains (**b**) in test (M-MIST+Er:YAG) and control (M-MIST) groups 12 months post op.

Figure 4a,b presents the frequency distribution of residual pockets and CAL gain after 1 year. In both groups, 5 out of 19 participants had a residual pocket PD ≥ 5 mm (26.3%). In the M-MIST+Er:YAG+Nd:YAG group, one pocket was shallowed to 2 mm (5.3%). There were no pockets shallower than 3 mm in the M-MIST group. In both groups, there were 2 patients with CAL gain ≥ 5 mm (10.5% of each group). However, in the control group, one patient lost CAL, and the second had no CAL gain. In the test group, only 1/19 patients had no CAL gain after 12 months.

Evaluation of radiographs at 12 months demonstrated statistically significant improvements in radiological defect depth in both groups and in radiological defect width in the M-MIST group. However, no significant differences were noted between groups. In the test group, RxD demonstrated a reduction of 1.38 mm, and in the control group, 1.34 mm. A slight decrease was also noted in the RxW, amounting to 0.14 mm in the test and 0.4 mm in the control group (Table 4).

## 4. Discussion

The results of the present study have shown that the effectiveness of intrabony defect treatment with M-MIST or M-MIST in combination with Er:YAG and Nd:YAG lasers is comparable. The values of plaque and bleeding indices indicate that the patients have undergone proper hygienic preparation and achieved an optimal level of inflammation control throughout the observation period. Both methods led to statistically significant improvements in clinical and radiological parameters. PD reduction was 2.95 mm in both groups, and CAL gain was slightly better in the M-MIST+Er:YAG+Nd:YAG group (3 mm) than in the M-MIST group (2.63 mm), but the difference was not significant. At 12 months postoperatively, radiographic defect depth reduction was very similar in both groups, that is, 1.38 mm in the test group and 1.34 mm in the control group, respectively. The radiographic defect width decrease was more pronounced in the control (0.4 mm) than in the test (0.14 mm) group. Interestingly, despite a similar frequency distribution of residual pockets and CAL gain only in test group one, a pocket was shallowed to 2 mm, and there was no attachment loss in any defects treated. The six-month results of our study, published previously, also indicate that the additional use of Er:YAG+Nd:YAG lasers does not improve the clinical and biochemical treatment outcomes [24]. The results presented in our study show that laser debridement is non-inferior compared to the conventional debridement using hand periodontal curettes and ultrasonic scalers for treatment of intrabony defects.

The results of our twelve-month observations correlate with other authors’ reports regarding minimally invasive periodontal surgery. One of its most pronounced advantages is the low incidence of pain and discomfort [22,23]. Of course, the operator’s experience is also important. All procedures were performed by one surgeon with over ten years of experience in regenerative procedures. Although the time required to perform the surgical procedure was not measured, the fact that no adverse events occurred indicates the high qualification and experience of the operator. The second important aspect is the incidence of recession, which is lower in the case of MIST procedures than in standard periodontal surgeries [23]. In our study, GR did not increase in the test group and only increased slightly (0.32 mm) in the control group. According to a systematic review that included 18 studies, the mean GR increase was 0.44 mm in minimally invasive surgical procedures [25]. Limiting the mesio-distal flap preparation and preserving the papilla enables recession reduction and better aesthetic outcomes, which is now essential. In our study, PD reduction after M-MIST and M-MIST with additional laser utilization was 2.95 mm, whereas it was 4.6 mm according to Cortellini [12], 3.5 mm according to Cosyn et al. [26], 3.82 mm in the M-MIST group according to Mishra [27], and 3.5 mm in the MIST group according to Ribeiro et al. [28]. Respectively, CAL gain in our study was 3 mm for the test and 2.63 mm for the control group, which can be compared with 4.5 mm in the study of Cortellini [12], 3.1 mm in the study of Cosyn et al. [26], 2.64 mm in the M-MIST group according to Mishra [27], and 2.8 mm in the MIST group according to Ribeiro et al. [28]. Our clinical results are closest to Mishra et al. and Ribeiro et al. outcomes. It should be emphasized, however, that Cortellini et al. in their trial utilized enamel matrix derivatives (EMD), and Cosyn et al. used a collagen-enriched bovine-derived xenograft, which could also affect the clinical parameters. Interestingly, there is evidence in the literature that the sole MIST procedure is as effective as MIST with biomaterials and membranes [29,30]. That is in opposition to traditional surgical approaches, where the utilization of regenerative materials has improved the clinical results of periodontal surgeries [31,32]. Promising clinical results of minimally invasive surgery are probably associated with a high incidence of primary flap closure that enables clot stability, space maintenance, and good flap perfusion [30,33]. Cortellini et al. stated that. M-MIST alone provided similar short- and long-term benefits as regeneration, at a lower cost. It was pointed out, however, that careful supportive periodontal care (SPC) is essential for long-term success [34]. The second important factor that influences the long-term stability of regenerated area is smoking [35,36]. The influence of smoking on periodontal treatment, both surgical and nonsurgical, has been highlighted in the literature. Lots of research has shown much worse clinical results expressed by inferior reduction in the probing depth and clinical attachment gains in smoking patients compared to non-smoking patients [37,38,39]. Smokers were not qualified for our study.

Lasers are promising devices in both periodontal treatment and periodontal reconstructive surgery. In the last systematic review concerning minimal invasiveness in the treatment of intraosseous defects, low-level laser biostimulation of the defect is recommended to favorably modulate the postoperative course of treatment [40]. This is the premise for using lasers not only to control the inflammation but also to provide stimulation with photonic energy. Different types of lasers are used in conjunction with periodontal surgical procedures. Er:YAG laser was used with open flap debridement with a benefit [41,42]. Surgical treatment of single-rooted teeth with Er:YAG laser was more effective according to PD reductions and CAL gains than conventional Widman flap surgery in 5 5-year follow-up [41]. A higher tendency for CAL gain was also observed in a short-term clinical trial comparing open flap surgery with and without Er:YAG laser [42]. Furthermore, the Er:YAG laser may be an alternative to root biomodification due to the irregularities in the root surface after its application and the effective removal of the smear layer, which potentially increases the growth and adhesion of cells and blood components [43]. Nd:YAG laser was also tested for the possibility of root conditioning instead of EDTA with EMD in intrabony defects, and did not improve the outcome [44]. But Nd:YAG laser is also used in the LANAP^®^ procedure, which is effective, and there is histological evidence for “cementum-mediated periodontal ligament attachment in the absence of a long junctional epithelium” [45,46]. A systematic review concerning the application of lasers in surgical therapy is prudent and points out that there is insufficient evidence for the adjunctive benefits of lasers in periodontal surgery [47].

Although our study was precisely performed, it has some limitations. The study was conducted in a single center, so the outcomes cannot be directly applied to other populations. No clinical intra data after 12 months and histological evidence are available, because there were no re-entry surgeries or histology performed. Further studies, planned as multicenter trials with larger sample sizes and undertaken with histological analysis, are needed to confirm the regeneration of periodontal tissues after proposed laser usage.

## 5. Conclusions

In conclusion, within the limits of this study, our results indicate that the use of Er:YAG and Nd:YAG lasers combined with the M-MIST procedure and the conventional M-MIST procedure provides comparable clinical and radiological treatment outcomes.

## Figures and Tables

**Table 1:** Characteristics of study participants and teeth included in the surgical procedures (clinical parameters in mm).

	No	Gender	Mean Age	Incisors/Canines/ Premolars/Molars	Defect Walls 3walls/2walls/1wall	Mean Intrasurgical Defect Depth	Mean Intrasurgical Defect Width
Test (M-MIST+Er:YAG+Nd:YAG)	19	12F/7M	47	3/3/12/1	5/11/3	4.03	2.58
Control (M-MIST)	19	9F/10M	43.5	3/1/9/6	3/15/1	4.34	2.95
significance		NS	NS	-	-	NS	NS

**Table 2:** Indices of oral hygiene (FMPS) and clinically visible inflammation (FMBOP) in test (M-MIST+Er:YAG) and control (M-MIST) groups at baseline and in a one-year follow-up.

	Basal	3 Months	6 Months	12 Month	*p* * (Changes in Time)
**FMPS**					
M-MIST+Er:YAG+Nd:YAG	10.79 ± 4.41	12.21 ± 6.28	12.23 ± 6.61	12.16 ± 6.35	NS
M-MIST	11.59 ± 4.67	14.36 ± 9.97	11.82 ± 7.33	9.91 ± 4.42	NS
*p* ** (between groups)	NS	NS	NS	NS	
**FMBOP**					
M-MIST+Er:YAG+Nd:YAG	10.71 ± 3.89	9.35 ± 5.02	11.60 ± 6.14	14.81 ± 7.43	NS
M-MIST	10.34 ± 4.58	10.32 ± 8.57	10.67 ± 7.59	9.69 ± 4.95	NS
*p* ** (between groups)	NS	NS	NS	*p* = 0.035	

*p* *—Wilcoxon pair test, *p* **—U Mann–Whitney test, NS—non-significant.

**Table 3:** Clinical parameters in test (M-MIST+Er:YAG) and control (M-MIST) groups at baseline and 12 months post-op.

	Basal	12 Month	*p* * (Changes in Time)	Basal Median	12 Month Median	Diff 0–12
**PD (mm)**						
M-MIST+Er:YAG+Nd:YAG	7.26 ± 1.44	4.31 ± 1.63	*p* = 0.0002	7 (6–11)	4 (2–9)	2.95 ± 1.31
M-MIST	7.15 ± 1.25	4.21 ± 1.43	*p* = 0.0002	7 (5–9)	4 (3–9)	2.95 ± 1.35
*p* ** (between groups)		NS				NS
**GR (mm)**						
M-MIST+Er:YAG+Nd:YAG	1.31 ± 1.34	1.26 ± 1.09	NS	1.5 (0–4)	1 (0–3)	0.05 ± 0.6
M-MIST	0.89 ± 1.19	1.21 ± 1.61	NS	0 (0–4)	1 (0–5)	−0.32 ± 1.00
*p* ** (between groups)		NS				NS
**CAL (mm)**						
M-MIST+Er:YAG+Nd:YAG	8.57 ± 2.16	5.57 ± 1.86	*p* = 0.0002	8 (6–15)	6 (2–9)	3.0 ± 1.57
M-MIST	8.05 ± 1.8	5.42 ± 2.77	*p* = 0.0008	8 (5–12)	4 (3–14)	2.63 ± 2.03
*p* ** (between groups)		NS				NS
**KT (mm)**						
M-MIST+Er:YAG+Nd:YAG	3.78 ± 1.78	3.73 ± 1.69	NS	4 (1–7)	4 (1–7)	0.05 ± 0.85
M-MIST	4.47 ± 1.38	4.10 ± 1.52	NS	4 (2–7)	4 (2–7)	0.37 ± 1.3
*p* ** (between groups)		NS				NS

*p* *—Wilcoxon pair test, *p* **—U Mann–Whitney test, NS—non-significant, diff—difference 0–12.

**Table 4:** Radiological defect depth (RxD) and width (RxW) in test (M-MIST+Er:YAG) and control (M-MIST) groups at baseline and 12 months post op.

	Basal	12 Month	*p* * (Changes in Time)	Basal Median	12 Month Median	Diff 0–12
**RxD (mm)**						
M-MIST+Er:YAG+Nd:YAG	4.42 ± 1.98	3.04 ± 1.75	*p* = 0.0007	3.6 (2.3–9.8)	2.6 (0–7.3)	1.38 ± 1.47
M-MIST	3.71 ± 1.22	2.37 ± 1.02	*p* = 0.0002	3.3 (2.2–6.3)	2.5 (0.8–4.3)	1.34 ± 1.08
*p* ** (between groups)		NS				NS
**RxW (mm)**						
M-MIST+Er:YAG+Nd:YAG	2.13 ± 0.83	1.99 ± 0.91	NS	2.2 (0.8–3.3)	2.4 (0–3.3)	0.14 ± 0.56
M-MIST	2.28 ± 1.04	1.88 ± 1.13	*p* = 0.006	2.2 (1.1–5.5)	1.6 (0.7–6)	0.4 ± 0.58
*p* ** (between groups)		NS				NS

*p* *—Wilcoxon pair test, *p* **—U Mann–Whitney test, NS—non-significant, diff—difference 0–12.

## Data Availability

The datasets used and/or analyzed during the current study are available from the corresponding author on reasonable request.

## References

[1] Chapple I.L.C., Mealey B.L., Van Dyke T.E., Bartold P.M., Dommisch H., Eickholz P., Geisinger M.L., Genco R.J., Glogauer M., Goldstein M. (2018). Periodontal health and gingival diseases and conditions on an intact and a reduced periodontium: Consensus report of workgroup 1 of the 2017 World Workshop on the Classification of Periodontal and Peri-Implant Diseases and Conditions. J. Periodontol..

[2] Papapanou P.N., Sanz M., Buduneli N., Dietrich T., Feres M., Fine D.H., Flemmig T.F., Garcia R., Giannobile W.V., Graziani F. (2018). Periodontitis: Consensus report of workgroup 2 of the 2017 World Workshop on the Classification of Periodontal and Peri-Implant Diseases and Conditions. J. Periodontol..

[3] Suvan J., Leira Y., Moreno F., Graziani F., Derks J., Tomasi C. (2020). Subgingival instrumentation for treatment of periodontitis: A systematic review. J. Clin. Periodontol..

[4] Herrera D., Matesanz P., Martín C., Oud V., Feres M., Teughels W. (2020). Adjunctive effect of locally delivered antimicrobials in periodontitis therapy: A systematic review and meta-analysis. J. Clin. Periodontol..

[5] Figuero E., Roldán S., Serrano J., Escribano M., Martín C., Preshaw P.M. (2020). Efficacy of adjunctive therapies in patients with gingival inflammation: A systematic review and meta-analysis. J. Clin. Periodontol..

[6] Sanz M., Herrera D., Kebschull M., Chapple I., Jepsen S., Berglundh T., Sculean A., Tonetti M.S., EFP Workshop Participants and Methodological Consultants (2020). Treatment of stage I–III periodontitis—The EFP S3 level clinical practice guideline. J. Clin. Periodontol..

[7] Stavropoulos A., Bertl K., Spineli L.M., Sculean A., Cortellini P., Tonetti M. (2021). Medium- and long-term clinical benefits of periodontal regenerative/reconstructive procedures in intrabony defects: Systematic review and network meta-analysis of randomized controlled clinical studies. J. Clin. Periodontol..

[8] Papapanou P.N., Tonetti M.S. (2000). Diagnosis and epidemiology of periodontal osseous lesions. Periodontology 2000.

[9] Kim C.K., Choi S.H., Kim T.S., Kaltschmitt J., Eickholz P. (2006). The infrabony defect and its determinants. J. Periodontal Res..

[10] Herrera D., Sanz M., Kebschull M., Jepsen S., Sculean A., Berglundh T., Papapanou P.N., Chapple I., Tonetti M.S., EFP Workshop Participants and Methodological Consultant (2022). Treatment of stage IV periodontitis: The EFP S3 level clinical practice guideline. J. Clin. Periodontol..

[11] Cortellini P., Prato G.P., Tonetti M.S. (1995). The modified papilla preservation technique: A new surgical approach for interproximal regenerative procedures. J. Periodontol..

[12] Cortellini P., Tonetti M.S. (2009). Improved wound stability with a modified minimally invasive surgical technique in the regenerative treatment of isolated interdental intrabony defects. J. Clin. Periodontol..

[13] Graziani F., Gennai S., Cei S., Cairo F., Baggiani A., Miccoli M., Gabriele M., Tonetti M. (2012). Clinical performance of access flap surgery in the treatment of the intrabony defect: A systematic review and meta-analysis of randomized clinical trials. J. Clin. Periodontol..

[14] Aljateeli M., Koticha T., Bashutski J., Sugai J.V., Braun T.M., Giannobile W.V., Wang H.L. (2014). Surgical periodontal therapy with and without initial scaling and root planing in the management of chronic periodontitis: A randomized clinical trial. J. Clin. Periodontol..

[15] Estrin N.E., Moraschini V., Zhang Y., Romanos G.E., Sculean A., Miron R.J. (2022). Combination of Nd:YAG and Er:YAG lasers in non-surgical periodontal therapy: A systematic review of randomized clinical studies. Lasers Med. Sci..

[16] Mizutani K., Aoki A., Coluzzi D., Yukna R., Wang C.Y., Pavlic V., Izumi Y. (2016). Lasers in minimally invasive periodontal and peri-implant therapy. Periodontology 2000.

[17] Ishikawa I., Aoki A., Takasaki A.A., Mizutani K., Sasaki K.M., Izumi Y. (2009). Application of lasers in periodontics: True innovation or myth?. Periodontology 2000.

[18] Abu-Ta’a M., Karameh R. (2022). Laser and Its Application in Periodontology: A Review of Literature. Open J. Stomatol..

[19] Al Asmari D., Alenezi A. (2025). Laser Technology in Periodontal Treatment: Benefits, Risks, and Future Directions—A Mini Review. J. Clin. Med..

[20] O’Leary T.J., Drake R.B., Naylor J.E. (1972). The plaque control record. J. Periodontol..

[21] Ainamo J., Bay I. (1975). Problems and proposals for recording gingivitis and plaque. Int. Dent. J..

[22] Cortellini P., Tonetti M.S., Lang N.P., Suvan J.E., Zucchelli G., Vangsted T., Silvestri M., Rossi R., McClain P., Fonzar A. (2001). The simplified papilla preservation flap in the regenerative treatment of deep intrabony defects: Clinical outcomes and postoperative morbidity. J. Periodontol..

[23] Cortellini P., Tonetti M.S. (2007). Minimally invasive surgical technique (M.I.S.T.) and enamel matrix derivative (EMD) in intrabony defects. (I) Clinical outcomes and intraoperative and post-operative morbidity. J. Clin. Periodontol..

[24] Dolińska E., Skurska A., Dymicka-Piekarska V., Milewski R., Pietruska M. (2024). Matrix metalloproteinase 9 (MMP-9) and interleukin-8 (IL-8) in gingival crevicular fluid after minimally invasive periodontal surgery with or without Er:YAG and Nd:YAG laser application. Antibiotics.

[25] Clementini M., Ambrosi A., Cicciarelli V., De Risi V., De Sanctis M. (2019). Clinical performance of minimally invasive periodontal surgery in the treatment of infrabony defects: Systematic review and meta-analysis. J. Clin. Periodontol..

[26] Cosyn J., Cleymaet R., Hanselaer L., De Bruyn H. (2012). Regenerative periodontal therapy of infrabony defects using minimally invasive surgery and a collagen-enriched bovine-derived xenograft: A 1-year prospective study on clinical and aesthetic outcome. J. Clin. Periodontol..

[27] Mishra A., Avula H., Pathakota K.R., Avula J. (2013). Efficacy of modified minimally invasive surgical technique in the treatment of human intrabony defects with or without use of rhPDGF-BB gel: A randomized controlled trial. J. Clin. Periodontol..

[28] Ribeiro F.V., Casarin R.C., Palma M.A., Júnior F.H., Sallum E.A., Casati M.Z. (2013). Clinical and microbiological changes after minimally invasive therapeutic approaches in intrabony defects: A 12-month follow-up. Clin. Oral Investig..

[29] Cortellini P., Tonetti M.S. (2011). Clinical and radiographic outcomes of the modified minimally invasive surgical technique with and without regenerative materials: A randomized-controlled trial in intra-bony defects. J. Clin. Periodontol..

[30] Liu S., Hu B., Zhang Y., Li W., Song J. (2016). Minimally invasive surgery combined with regenerative biomaterials in treating intra-bony defects: A meta-analysis. PLoS ONE.

[31] Needleman I., Tucker R., Giedrys-Leeper E., Worthington H. (2005). Guided tissue regeneration for periodontal intrabony defects—A Cochrane systematic review. Periodontology 2000.

[32] Nibali L., Koidou V.P., Nieri M., Barbato L., Pagliaro U., Cairo F. (2020). Regenerative surgery versus access flap for the treatment of intra-bony periodontal defects: A systematic review and meta-analysis. J. Clin. Periodontol..

[33] Retzepi M., Tonetti M., Donos N. (2007). Gingival blood flow changes following periodontal access flap surgery using laser Doppler flowmetry. J. Clin. Periodontol..

[34] Cortellini P., Cortellini S., Bonaccini D., Tonetti M.S. (2022). Modified minimally invasive surgical technique in human intrabony defects with or without regenerative materials—10-year follow-up of a randomized clinical trial: Tooth retention, periodontitis recurrence, and costs. J. Clin. Periodontol..

[35] Silvestri M., Rasperini G., Milani S. (2011). 120 infrabony defects treated with regenerative therapy: Long-term results. J. Periodontol..

[36] Patel R.A., Wilson R.F., Palmer R.M. (2012). The effect of smoking on periodontal bone regeneration: A systematic review and meta-analysis. J. Periodontol..

[37] Tomasi C., Wennström J.L. (2009). Full-mouth treatment vs. the conventional staged approach for periodontal infection control. Periodontology 2000.

[38] Labriola A., Needleman I., Moles D.R. (2005). Systematic review of the effect of smoking on nonsurgical periodontal therapy. Periodontology 2000.

[39] Heasman L., Stacey F., Preshaw P.M., McCracken G.I., Hepburn S., Heasman P.A. (2006). The effect of smoking on periodontal treatment response: A review of clinical evidence. J. Clin. Periodontol..

[40] Simonelli A., Severi M., Trombelli L., Farina R. (2023). Minimal invasiveness in the surgical treatment of intraosseous defects: A systematic review. Periodontology 2000.

[41] Gaspirc B., Skaleric U. (2007). Clinical evaluation of periodontal surgical treatment with an Er:YAG laser: 5-year results. J. Periodontol..

[42] Sculean A., Schwarz F., Berakdar M., Windisch P., Arweiler N.B., Romanos G.E. (2004). Healing of intrabony defects following surgical treatment with or without an Er:YAG laser. J. Clin. Periodontol..

[43] Lavu V., Sundaram S., Sabarish R., Rao S.R. (2015). Root surface biomodification with erbium lasers—A myth or a reality?. Open Dent. J..

[44] Dilsiz A., Canakci V., Aydin T. (2010). The combined use of Nd:YAG laser and enamel matrix proteins in the treatment of periodontal infrabony defects. J. Periodontol..

[45] Yukna R.A., Carr R.L., Evans G.H. (2007). Histologic evaluation of an Nd:YAG laser-assisted new attachment procedure in humans. Int. J. Periodontics Restor. Dent..

[46] Nevins M.L., Camelo M., Schupbach P., Kim S.W., Kim D.M., Nevins M. (2012). Human clinical and histologic evaluation of laser-assisted new attachment procedure. Int. J. Periodontics Restor. Dent..

[47] Behdin S., Monje A., Lin G.H., Edwards B., Othman A., Wang H.L. (2015). Effectiveness of laser application for periodontal surgical therapy: Systematic review and meta-analysis. J. Periodontol..

